# On the Coupling of Two Models of the Human Immune Response to an Antigen

**DOI:** 10.1155/2014/410457

**Published:** 2014-07-22

**Authors:** Bárbara de M. Quintela, Rodrigo Weber dos Santos, Marcelo Lobosco

**Affiliations:** Laboratory of Computational Physiology and High-Performance Computing (FISIOCOMP), Graduate Program in Computational Modeling, UFJF, Rua José Lourenço Kelmer s/n, Campus Universitário, Bairro São Pedro, 36036-900 Juiz de Fora, MG, Brazil

## Abstract

The development of mathematical models of the immune response allows a better understanding of the multifaceted mechanisms of the defense system. The main purpose of this work is to present a scheme for coupling distinct models of different scales and aspects of the immune system. As an example, we propose a new model where the local tissue inflammation processes are simulated with partial differential equations (PDEs) whereas a system of ordinary differential equations (ODEs) is used as a model for the systemic response. The simulation of distinct scenarios allows the analysis of the dynamics of various immune cells in the presence of an antigen. Preliminary results of this approach with a sensitivity analysis of the coupled model are shown but further validation is still required.

## 1. Introduction

Systems biology is an emerging interdisciplinary area of science that advocates a distinct perspective on the study of biological phenomena, particularly focusing on understanding a system's structure and its dynamics [1]. The systems biology approach often involves the use of mathematical and computational techniques in the development of mechanistic models that describes complex interactions in biological systems.

One complex biological system that can benefit from the systems biology approach is the immune system (IS). The IS is composed by a large number of cells, molecules, tissues, and organs that form a complex network that interact with each other in order to protect the body against pathogenic agents [2]. The IS of vertebrates is composed by three layers of defense: (a) the physical barriers; (b) innate IS; and (c) the adaptive IS.

The physical barriers are composed by the skin and mucous membranes that form a shield against the pathogenic agents. If this shield is crossed by pathogens, they encounter cells and molecules of the innate IS, such as proteins of the complement system and macrophages, that immediately develop a response to them. The macrophages phagocyte the pathogens and produce proteins called cytokines that signal to other innate cells that their help is needed. Recruitment of more innate cells is essential for effective control of infections [3]. Some of the innate cells such as the macrophages and dendritic cells act as antigen presenting cells (APCs), playing a pivotal role on activating the third layer of defense, which is the adaptive IS response. B and T lymphocytes are some of the main cells of this third layer response. The presence of these cells is extremely important because they can adapt to deal with almost any invader. B cells in its plasma form secrete antibodies. Antibodies bind to pathogens, which turns the latter more susceptible to phagocytosis (in a process called opsonization). Three main types of T cells are known: (a) killer T cells (also known as cytotoxic lymphocytes), (b) helper T cells, and (c) regulatory T cells. Killer T cells induce infected cells to commit suicide, in a process called apoptosis; helper T (Th2) cells produce cytokines and help priming B cells; and regulatory T cells act in the regulation of the response, although the complete process is still unclear [2].

A large number of works have proposed models to describe the IS [4–13]. A great introduction to previous models of the IS is available in the work of Perelson and Weisbuch [14] and more recently the state of the art on representing the IS was presented by Narang et al. [15].

Computational models of the adaptive IS are very often developed using pure mathematical tools, such as ordinary differential equations (ODEs), to describe the behavior of its components and their relationships, although other tools, such as system dynamics [16, 17], cellular automata [18–24], agent-based systems [25–36], and complex adaptive systems [37], are also used. Some works focus only on modeling the innate IS [38–40], which is responsible for activating the adaptive response.

The use of more than one approach to model the immune response is not novel and there are different works that consider differential equations together with cellular automata [23], agent-based Systems [41], and system dynamics [16]. And also, the assumption of different scales of the immune system was already considered by Kirschner [33]. However, none of the previous approaches so far represented the immune response considering the spatial features such as, cellular movement, diffusion, and chemoattraction modeled with PDEs and the dynamics of antibody activation modeled with ODEs, which is the approach used in this work. Differential equations were chosen due to the advantage of dealing with distinct scales, in comparison with other approaches, and the possibility of numerical analysis of the model.

A previous work presented a mechanistic computational model of the innate immune response to a general pathogen [42]. This pathogen is represented by lipopolysaccharide (LPS) that is present in the outer membrane of Gram-negative bacteria. That model represents the behavior of the main defense cells, such as macrophages, and molecules, such as proinflammatory cytokines (TNF-*α* and IL-8) and anti-inflammatory cytokines (IL-10). A set of PDEs was used to reproduce important phenomena such as the temporal order of cells arriving at the local of infection, the production of proinflammatory and anti-inflammatory cytokines, and the chemotaxis phenomenon. The model has been extended (a) to allow the use of a three-dimensional domain in order to better represent the site of infection and (b) to use parallel programming techniques to guarantee a reasonable simulation time [43]. This work extends our previous models [42, 43], enabling the innate IS to activate the adaptive one. The main contribution of this work is a mathematical model that reproduces the dynamics of both innate and adaptive IS, coupling for this purpose models of different nature and scales. The adaptive model chosen to be coupled with the innate model consists of a set of equations, based on the mathematical model of pneumonia which is described in [44]. Besides, the coupled model represents a more complete scenario: the dynamics of the cells and molecules inside the tissue as well as the communication through lymph and blood vessels with the nearest lymph node. We must stress that the main contribution of this work is the new way used to couple models of distinct scales and aspects of the immune system, represented by ODEs and PDEs. In this way, both models used to represent the innate and the adaptive immune system could be replaced by other models with slight modifications. In fact, several recent models represent the dynamics of acute inflammatory response in the lung [45–49]. The model proposed by [44] was chosen due to the availability of all parameters needed to implement the model.

The interest in modeling spatial features of the immune response is due to the increasing availability of noninvasive imaging techniques mainly on the last decade [50]. Besides, the spatial information of an individual could be provided at any time to validate the* in silico* models. A few examples of recent works on noninvasive imaging in immunology are [51–55]. Thus, an important aspect considered in choosing pneumonia as an example to illustrate the new coupling technique and why to use PDEs is the fact that this disease causes damages in the tissue that can be observed by medical imaging techniques. The simulation of the coupled model can generate as a result an image that represents the damage caused by bacteria to the local tissue, in this example the lung (alveolar space).

Related works on coupling models of the IS deal with different scales to represent the trigger for innate response and activation of acquired response. At a molecular level [56] used coupled ODEs models to understand the behavior of the proteins involved in the process of antigenic presentation. At a cellular level there are works that couple models employing agent-based systems with ODEs [31, 33, 57], or with system dynamics [16], while others use only PDEs [39, 58], only ODEs [45, 59], or DDEs [60] to achieve this purpose. A similar approach using only ODEs to represent tuberculosis dynamics can be found in [61]. The model proposed in this work uses both PDEs and ODEs to describe the entire dynamics. This is, for the best of our knowledge, the first work that couples PDEs and ODEs to describe the dynamics of innate and adaptive IS into a piece of tissue, including the activation of the adaptive IS by the innate IS.

This work is organized as follows.  Section 2 presents the biological model used in this work and the coupling of the mathematical models. The IS model presented in this work was simplified when compared to our previous models [42, 43]. The complete model was not used in order to focus on the integration of the two different models: (a) local tissue and (b) lymph nodes.  Section 3 presents its computational implementation. The results obtained by the models, the discussion, and a sensitivity analysis of the coupled model are presented in Section 4. Finally, Section 5 draws our conclusions and present our future works.

## 2. Materials and Methods

### 2.1. Biological Model

**Figure 1:**
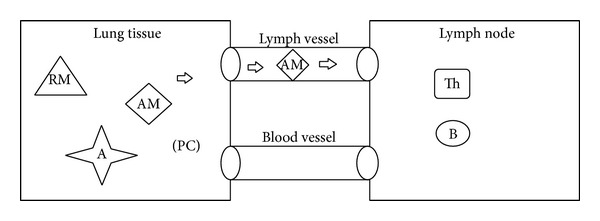
Communication between local tissue and lymph node; activation of resting macrophages (RM) and migration of activated macrophages (AM) to the lymph node.

According to [44], there are several microorganisms that could be etiological agents of destructive pneumonia. However, the inflammatory and destructive process in lung tissue cannot be started unless there is a malfunctioning of local and general defense mechanisms [62]. The multiplication of microorganisms occurs between 10 and 14 days and causes the disease which, in case of recovery, ends as a result of destruction of bacteria by antibodies, macrophages, and neutrophils. Initially, we consider a simplified scenario where there are only resting macrophages located in the tissue (as an example we chose the lung tissue) and T- and B-lymphocytes in the lymph nodes. After the injection of an antigen (A), those resting macrophages (RM) that encounter an antigen become activated macrophages (AM) and start producing proinflammatory cytokines (PC) to recruit other immune cells to the site of infection. Activated macrophages act as antigen presenting cells (APCs) migrating to the closest lymph node through lymph vessels (Figure 1) and exhibiting the antigen to the lymphocytes which initiates the activation and differentiation of T-lymphocytes into T-helper 2 lymphocytes (Th2) and activation and differentiation of B-lymphocytes into plasma cells.

**Figure 2:**
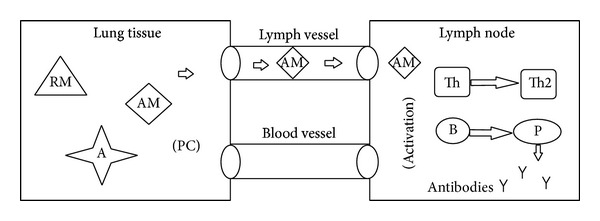
Activated macrophage (AM) stimulate lymphocytes by antigen presenting process; T cell differentiate in T-helper 2 (Th2) and B cell differentiate in plasma cell (*P*) which produces antibodies (*Y*).

**Figure 3:**
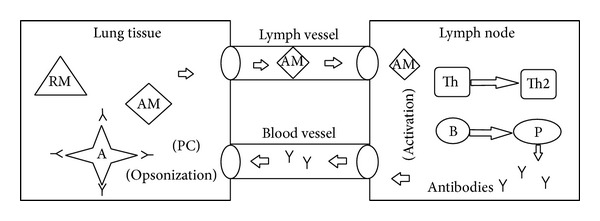
Antibodies (*Y*) migrate to the lung tissue through blood vessel and opsonize the antigen (*A*).

Plasma cells are mass producers of antibodies (Figure 2). Those antibodies head to the local of the infection in the lung tissue through blood vessels. As soon as they reach an antigen they do what is called opsonization of the antigen, the process by which an antigen is marked for ingestion and destruction by a phagocyte (Figure 3) [63].

### 2.2. Coupling of Models

Marchuk uses DDEs to model the processes involved in pneumonia [44]. In this work, some assumptions are made to model that scenario.

- We do not consider the delay on biological processes such as T lymphocyte clonal expansion and antibodies production and release.
- We consider only the temporal behavior of the cells inside the lymph node and the spatiotemporal behavior of the cells in the lung tissue. Thus, we expect to visualize the damage caused to the lung parenchyma.

In order to represent the immune response, PDEs based on a previous model of the innate response [42] have been employed to model the spatial and temporal behavior of the following components:

 
*S. aureus* bacteria (*A*);
 resting macrophages (*M*_*R*_);
 activated macrophages (*M*_*A*_);
 specific antibodies (*F*).

Moreover, ODEs represent the cascade activation of the lymphocytes leading to the production and release of antibodies in the lymph node (LN), which is a simplification of the model in [44]:

 T-lymphocytes (*T*);
 B-lymphocytes (*B*);
 plasma cells (*P*);
 antibodies (*F* ^*L*^).

Macrophages act like APCs, activating the adaptive IS and the production and release of antibodies. The presentation and the later presence of antibodies in the infection site are only possible due to the coupling of the two distinct models presented herein. We assumed that the communication between the alveolar tissue and the nearest LN is guaranteed by the presence of lymph and blood vessels.

The linkage between the local and the systemic response is achieved by representing the APCs and the antibodies in both models. There is a PDE equation to model the activated macrophage behavior while they are inside the tissue and an ODE to model their concentration inside the LN. The flux of activated macrophages from the tissue to the LN involves the numerical integration of the macrophages in the tissue. The same style of approximation was adopted to model the migration of antibodies from the LN to the tissue. The PDEs based model and the ODEs based model are shown in the next subsection.

It must be stressed that pneumonia was chosen as an example to illustrate the framework proposed in this paper to couple models of different nature and scales; other models could be chosen to represent different infection processes caused by other diseases.

The general framework for the coupling of models proposed herein could be summarized as follows.

1. Selection of two distinct models of the immune response: one related to a local response and other related to a systemic one.
2. Identification of common or related variables among the chosen models, for example, the variables representing cells that migrate from one location to another during the response, and therefore act as APCs. If no common or related variable is found, a relation must then be created by adapting the models to the coupling as stated in the next step.
3. Implementation of necessary adjustments to the set of equations. This step could involve changes on the equations which must represent the flux between two different locations; for example, there must be a term to represent the flux of antibodies leaving the LN and another one representing the arrival of them in the tissue. Those terms do not necessarily exist on the original models that are being coupled. The units of measurement must also be considered within this step. If necessary, conversions should be made to the units of the parameters to keep the correctness of the coupled model.
4. Simulate the coupled model and validate the obtained results.

We expect to gain insights on the immune system response to distinct pathogenic agents with the coupling of models. It follows an example of the coupling of models to represent the immune response to* S. aureus* causing pneumonia.

### 2.3. PDEs Model

All the PDEs are modeled considering homogeneous Neumann boundary conditions.

#### 2.3.1. *S. aureus* Bacteria (*A*)

Equation (1) depicts the model for the* S. aureus* bacteria. The first term of (1) models the replication of bacteria at a rate *β*_*A*_ and carrying capacity is given by *k*_*a*_. The second term gives its natural decay by the coefficient *μ*_*A*_. Its engulfment by macrophages and other nonspecific defense cells is represented by the third and fourth terms of the same equation: *λ*_*MR*_ is the rate that accounts for the activation of macrophages and *λ*_*MA*_ is the destruction rate of bacteria by active macrophages.

(1) ∂A∂t=βAA(1−AkA)−μAA−λMRMRA−λMAAMA −λAF ∣ MRAFMR−λAF ∣ MAAFMA+DAΔA,A(x0,y0,z0,0)=A0,  ∂A∂t(·,t)|∂Ω=0.

The opsonization process for further phagocytosis in shown in the 5th and 6th terms; *λ*_*AF*∣*MR*_ represents the rate for destruction of opsonized bacteria by resting macrophages and *λ*_*AF*∣*MA*_ represents the rate for destruction by activated macrophages. The variable *F* is the amount of antibodies in the tissue modeled by (4) which is an important part of the coupling of models. The last term of this equation represents the diffusion of the bacteria in the lung tissue where *D*_*A*_ is the bacteria diffusion coefficient. Initially there is an injection of antigen only at one small part in the center of the tissue and it is assumed that there is no flux through the borders.

#### 2.3.2. Macrophages (*M*_*R*_, *M*_*A*_)

Macrophages are represented in two distinct states: resting (*M*_*R*_) and activated (*M*_*A*_) states. Initially, there are only resting macrophages in the tissue and they become activated after exposure to antigens (*A*). In the activated state, they play an important role in presenting and stimulating specific defense cells located in the LN.

Equation (2) represents the concentration of resting macrophages in the alveolar tissue, in which the first term accounts for their natural decay, the second term represents their activation, the third term represents the flux of resting macrophages entering the tissue from the blood, and the last one is the diffusion term. The natural decay rate is given by *μ*_*MR*_, *γ*_*MA*_ is the rate in which resting macrophage becomes active and *D*_*M*_*R*__ is the resting macrophage diffusion coefficient. Consider

(2) ∂MR∂t=−μMRMR−γMAMRA +αMRθBV(x,y,z)(MR∗−MR)+DMRΔMR,MR(x,y,z,0)=MR0,  ∂MR∂t(·,t)|∂Ω=0.

The flux of macrophages entering the tissue depends on the localization of the blood vessels in the tissue. In order to represent that behavior *α*_*M*_*R*__ represents the rate of migration and *θ*_BV_ is a function that is equal to 1 where volumes are in contact with blood vessels and 0 otherwise.

Equation (3) represents the concentration of activated macrophages in the alveolar tissue after encountering antigens. Again, the first term models their natural decay, at a rate of *μ*_*MA*_, the second term models their activation at a rate of *γ*_*MA*_, and the third one is the diffusion term, at a rate of *D*_*MA*_. Consider

(3) ∂MA∂t=−μMAMA+γMAMRA+DMAΔMA −αMθLV(x,y,z)(MA−MAL),MA(x,y,z,0)=MA0,  ∂MA∂t(·,t)|∂Ω=0.

The last term of (3) models the connection between the activated macrophages in the tissue and the concentration that migrates to the nearest LN to act as APCs. It represents the flux of *M*_*A*_ between the local alveolar tissue and the LN through the lymph vessels. In this equation, *M*_*A*_^*L*^ is the macrophage concentration in the LN, which dynamics is described by (5) and *θ*_LV_ is a function that is equal to 1 if the volumes are in contact with lymph vessels and 0 otherwise.

It is assumed that in the beginning of the simulation there are only resting macrophages over the tissue and there is no flux through the borders.

#### 2.3.3. Antibodies (*F*)

Equation (4) describes the antibody mechanics within the lung tissue. The first and second terms represent the antibodies consumption to defeat bacteria in the opsonization process, at rates depending on the state of the phagocyte cell: *λ*_*FA*|*MR*_ for resting macrophages and *λ*_*FA*|*MA*_ for activated macrophages. The third term models the diffusion process of antibodies in the tissue, at a rate of *D*_*F*_, and the last term describes the flux of antibodies between the LN and the tissue, at a rate of *α*_*F*_, in which *F* ^*L*^ is the concentration of antibodies released by plasma cells in the LN (11). Consider

(4) ∂F∂t=−λFA|MRFAMR−λFA|MAFAMA +DFΔF+αFθBV(x,y,z)(FL−F),F(x0,y0,z0,0)=F0,  ∂F∂t(·,t)|∂Ω=0.

The last term of (4) is part of the model coupling and was added to the PDEs model to make the connection between the antibodies released in the LN and their migration through the blood vessels to the infection site. It is assumed that there is no flux through the borders.

### 2.4. ODEs Model

In the ODEs model the cellular homeostasis is guaranteed by the addition of an equilibrium term in the equations corresponding to the adaptive IS. The idea is to preserve the minimum amount of adaptive IS cells in the body [44].

#### 2.4.1. Macrophages (*M*_*A*_^*L*^)

In order to perform the coupling, the antigen presentation needed to be represented as a trigger to the acquired response. The concentration of active macrophages inside the LN which migrated from the tissue was modeled by (5). This equation represents the active macrophages which migrated from the local alveolar tissue to the LN through the lymph vessels, at a rate of *α*_*M*_

(5) $$\begin{array}{c} \frac{dM_{A}^{L}}{dt}=\alpha_{M}\left(M_{A}^{T}-M_{A}^{L}\right)\frac{V_{\text{LV}}}{V_{\text{LN}}},\\ M_{A}^{L}\left(0\right)=M_{A_{0}} \end{array}$$

in which *V*_LN_ is the assumed volume of the LN and *V*_LV_ is the integral of the volumes where there is contact with lymph vessels given by

(6) $$\begin{array}{c} V_{\text{LV}}=\int_{\Omega }\theta_{\text{LV}}\left(x,y,z\right)d\Omega . \end{array}$$

The average concentration of active macrophages in the tissue (*M*_*A*_^*T*^) was calculated by the integration of the values of active macrophages within the domain of simulation in contact with lymph vessels and is described by

(7) $$\begin{array}{c} M_{A}^{T}=\frac{1}{V_{\text{LV}}}\int_{\Omega }\theta_{\text{LV}}\left(x,y,z\right)M_{A}d\Omega , \end{array}$$

where Ω represents the volume of the whole tissue simulated.

#### 2.4.2. T-Lymphocytes (*T*)

The T-helper lymphocytes are stimulated by active macrophages in the LN and play an important role in the activation of B-lymphocytes and plasma cells to start the production of specific antibodies against antigens. The first part of (8) represents the activation of Th2 cells, with its clonal expansion leading to the appearance of new cells. *b*_*T*_ is the rate for the stimulation of Th2 cells and *ρ*_*T*_ is the number of descendants Th2 cells created by single division. The second term represents the expenditure of Th2 cells to stimulate B cells, at a rate of *b*_*p*_. Finally, the third term models the maintenance of the homeostasis in absence of antigenic stimulation

(8) $$\begin{array}{c} \frac{dT}{dt}=b_{T}\left(\rho_{T}TM_{A}^{L}-TM_{A}^{L}\right)-b_{p}M_{A}^{L}TB+\alpha_{T}\left(T^{*}-T\right),\\ T\left(0\right)=T_{0} \end{array}$$

in which T* is the steady state value of the concentration of T-helper cells.

#### 2.4.3. B-Lymphocytes (*B*)

After B-lymphocytes cells have been stimulated by T cells and active macrophages in the LN, they start to proliferate and turn into plasma cells. Their proliferation is represented by the first term of (9), in which *b*_*p*_^*b*^ is the rate for the stimulation of B cells and *ρ*_*B*_ is the number of new B cells as an outcome of the stimulation. Again, the second term shows the maintenance of the homeostasis in the absence of antigenic stimulation

(9) $$\begin{array}{c} \frac{dB}{dt}=b_{p}^{b}\left(\rho_{B}TM_{A}^{L}-TM_{A}^{L}B\right)+\alpha_{B}\left(B^{*}-B\right),\\ B\left(0\right)=B_{0} \end{array}$$

in which B* is the steady state value for the concentration of B cells.

#### 2.4.4. Plasma Cells (*P*)

The plasma cells are generated from stimulated B cells, T-cells, and active macrophages in the LN and are the cells that release antibodies against the specific antigen that was presented by the APCs

(10) $$\begin{array}{c} \frac{dP}{dt}=b_{p}^{p}\left(\rho_{P}TM_{A}^{L}B\right)+\alpha_{P}\left(P^{*}-P\right),\\ P\left(0\right)=P_{0}. \end{array}$$

The first term of (10) describes the generation and maturation of plasma cells from stimulated B cells, in which *b*_*p*_^*p*^ is the rate for the stimulation of plasma cells and *ρ*_*P*_ is the number of new plasma cells. The last term is the maintenance of the homeostasis in which P* is the steady state concentration of plasma cells.

#### 2.4.5. Antibodies (*F* ^*L*^)

Antibodies released by plasma cells in the LN are represented by

(11) $$\begin{array}{c} \frac{dF^{L}}{dt}=\rho_{F}P-\left(\alpha_{F}\theta_{\text{BV}}\left(x,y,z\right)\left(F^{L}-F^{T}\right)\right)\frac{V_{\text{BV}}}{V_{\text{LN}}},\\ F^{L}\left(0\right)=F_{0} \end{array}$$

in which the first term describes the production of antibodies, at rate *ρ*_*F*_, by plasma cells and the last term represents the connection between the two models (PDEs and ODEs). The last term of (11) describes the flux of antibodies between the LN and the tissue, at a rate of *α*_*F*_, on volumes with contact with the blood vessels given by *θ*_BV_. *F* ^*T*^ is the average number of antibodies in the tissue described by

(12) $$\begin{array}{c} F^{T}=\frac{1}{V_{\text{BV}}}\int_{\Omega }\theta_{\text{BV}}\left(x,y,z\right)F\;d\Omega , \end{array}$$

where Ω is the tissue domain and *V*_BV_ is the integral of the volumes where there is contact with blood vessels given by

(13) $$\begin{array}{c} V_{\text{BV}}=\int_{\Omega }\theta_{\text{BV}}\left(x,y,z\right)d\Omega . \end{array}$$

### 2.5. Initial Conditions and Parameters

The initial conditions for the PDEs model which describe the process of formation of inflammatory site are shown in Table 1.

It was considered that initially only a small portion of the tissue had the presence of antigens and the domain of simulation was 10 mm^3^. This initial injection of antigen was represented in the center of the hexahedral domain of simulation (between 3 mm and 7 mm over the axes). Initially it is also considered the presence of macrophages in its resting state equally distributed over the tissue.

Tables 2, 3, and 4 present the set of parameters used in the simulations. Almost all parameters used in the simulation were obtained in Marchuk [44] applying the necessary unit conversions and some fitting to the coupled model. The only exceptions are the diffusion coefficients *D*_*M*_*R*__, *D*_*M*_*A*__, and *D*_*F*_ which were estimated by Pigozzo et al. [42] and the diffusion coefficient of bacteria *D*_*A*_ based on the work of Haessler and Brown [62].

## 3. Implementation

The numerical method employed to solve the mathematical model was the finite difference method, a method commonly used in the numeric discretization of PDEs. The finite difference method is a method of resolution of differential equations that is based on the approximation of derivatives with finite differences [64, 65].

Our implementation is based on the finite difference method for the spatial discretization and the explicit method for the time evolution with an upwind scheme for the convective term of the equations. The upwind scheme discretizes the hyperbolic part of the PDEs using a bias for the flux direction given by the signal of the characteristic speeds. The upwind scheme uses an adaptive or solution-sensitive stencil to precisely simulate the direction of information propagation.

Below there is an example of a finite difference operator used in the discretization of the Laplace operator that simulates the diffusion phenomenon in 3D:

(14) DO(∂2O(x,y,z)∂x2+∂2O(x,y,z)∂y2+∂2O(x,y,z)∂z2)≈DO∗((o[x+1,y,z]−2∗o[x,y,z]+o[x−1,y,z])×(ΔX2)−1) +DO∗((o[x,y+1,z]−2∗o[x,y,z]+o[x,y−1,z])×(ΔY2)−1) +DO∗((o[x,y,z+1]−2∗o[x,y,z]+o[x,y,z−1])×(ΔZ2)−1).

In (14), *O* represents the discretization of some types of cells, such as resting and activated macrophages; *D*_*O*_ is the diffusion coefficient of these populations of cells, *x*, *y*, and *z* are the position in the space, and Δ*X*, Δ*Y*, and Δ*Z* are the space discretization.

The code was implemented using the C programming language and it was considered a 10 × 10 uniform grid with space discretization of Δ*x* = Δ*y* = Δ*z* = 0.1 representing 10 mm^3^ of tissue. The time step for both ODEs and PDEs is Δ*t* = 10^−4^ and the set of ODEs was solved with explicit Euler method.  Algorithm 1 shows the general implementation of the coupling of models in which *iterPerDay* = 10^4^, *numDays* depends on the scenario simulated—for the coupled model it was considered equal to 30. *A* is the amount of antigens still present in the tissue. The simulation finishes when its value is less than or equal to a given threshold value, in this case *tol* = 10^−6^.

An effort to parallelize the coupling of models is still in progress [43] and does not rely in the scope of this paper. More information about the implementation of the PDEs can be obtained in [40].

## 4. Results and Discussion

To show the importance of immune cells, molecules, and processes in the dynamics of the immune response and also to validate the coupling of models, a set of simulations were performed under distinct scenarios. The simulations start with a simple scenario were the cells of the IS are not considered (Case 1). Aiming to analyze the importance of the antibodies to the elimination of bacteria, the response is represented firstly by the simulation of only the innate cells (Case 2) and then with the complete model including activation of the lymphocytes, production, and migration of antibodies to the tissue (Case 3). A sensitivity analysis was performed in order to evaluate the behavior of the coupled mathematical model.

### 4.1. Case 1: Antigen Behavior

The purpose of this case is to show the diffusive term in the antigen Equation (1).

**Figure 4:**
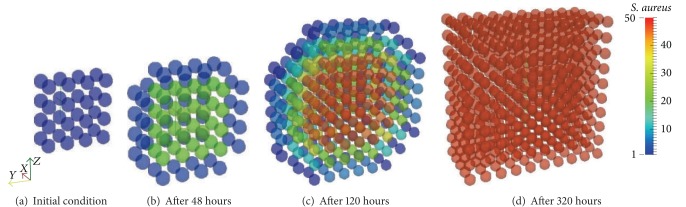
Initial condition and diffusion of the antigens at a 20-day simulation limited only by available space. After approximately 10 days of simulation the whole domain is filled with antigens.

Initially, the antigen is injected only in the middle portion of the three-dimensional domain (Figure 4(a)). All images in Figure 4 show a cut view of the volume along the *x*-axis in order to better visualize both the initial condition and the diffusion of the antigens. The simulation shows that, without the immune system cells, after a few hours the antigen starts to spread over the domain because of the diffusion (Figure 4(b)). Furthermore, the replication can also be observed with the increase of the population of* S. aureus* (Figures 4(c) and 4(d)). Before the end of the 20-day simulation it can be observed that there are antigens all over the domain in considerable amounts. In this case only (1) was simulated without considering any kind of response. The cubic domain was sliced in half to improve the visualization.

**Figure 5:**
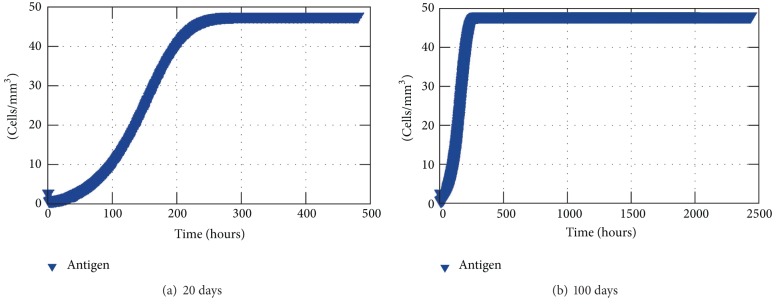
Antigen average concentration inside the tissue without any response.

Figures 5(a) and 5(b) show the logistical growth of antigen limited only by the space available, respectively, during 20 and 100 days. It can be observed that after 10 days the antigen reaches the maximum amount the simulated tissue could carry, which is assumed to be 50 antigens per cubic millimeter.

### 4.2. Case 2: Innate Response to an Antigen

**Figure 6:**
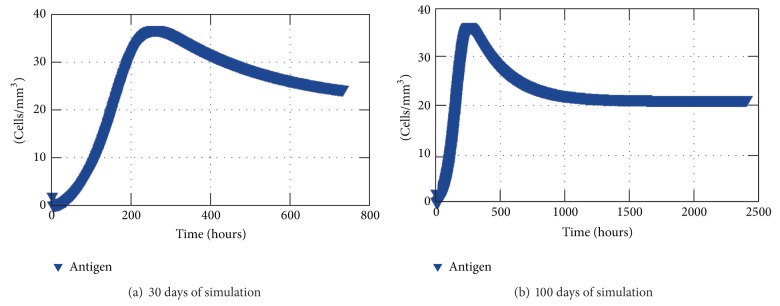
The results show the average of antigen in each mm^3^ of the tissue restrained only by the innate response.

Initially, without considering the trigger of the acquired response, the bacteria* S. aureus* increases for days held only by macrophages and the available space, which is assumed to contain at most 50 antigens per cubic millimeter. But after this initial period of time, macrophages are capable of restraining the antigen growth. However, they are not able to eliminate them completely. (Figure 6(a)).

**Figure 7:**
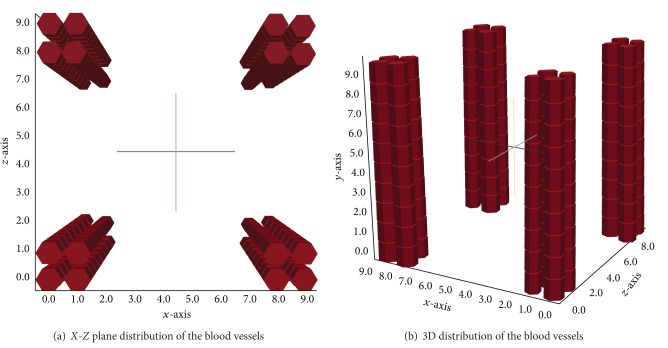
Scheme representing the position of the blood vessels on the edges of the tissue simulated.

Firstly, innate response to an antigen during 30 days was simulated (Figure 6(a)). According to the results shown by Figure 6(a), one can see that the amount of antigen increases until approximately 10 days (240 hours) and then decreases slowly. However, to understand what happens after those days that seemed to lead to an elimination of the antigen another test was performed for 100 days that showed that the initial amount of antigen injected in the tissue and the rates in which the innate immune cells arrive from the blood the antigen are not eliminated but stay on a chronic equilibrium state (Figure 6(b)). Throughout the simulations, the macrophages in the resting state come from the blood vessels which were positioned in the corners of the cubic domain along the *y*-axis (Figures 7(a) and 7(b)).

**Figure 8:**
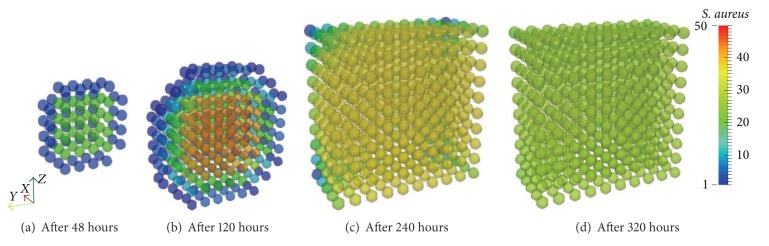
Antigen diffusion during 30 days of simulation limited by the presence of macrophages. The macrophages are capable of restraining the antigen growth but they are not able to defeat this initial amount.

The set of Figures 8(a)–8(d) show the spread of the antigen over the tissue in a 10 mm^3^ domain. Again, in order to better visualize the results the volume was cut along its *x*-axis. The initial amount of antigens was injected in the central portion of the tissue simulated (same used for Case 1) and it can be seen that the replication of the antigen and the absence of the acquired response lead to the spread of the antigen over the tissue restrained by the macrophages. The cubic domain was sliced in half to improve the visualization.

**Figure 9:**
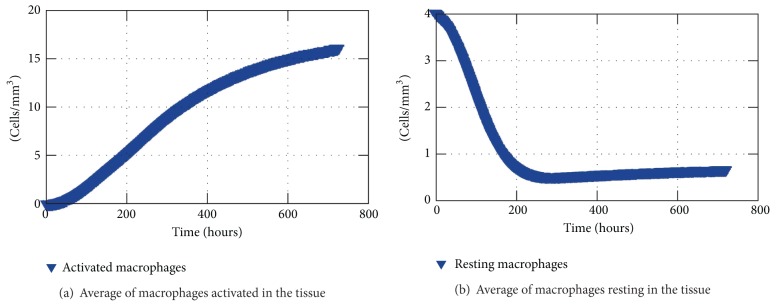
Macrophages in the tissue over 30 days of simulation.

Figures 9(a) and 9(b) depict the averages of macrophages both resting and activated in the tissue, respectively, over 30 days of simulation. The state changing from resting to activated can be observed as the resting population decreases while the activated macrophage population increases, though they are not exactly opposite curves due to distinct phagocytosis and decay rates.

### 4.3. Case 3: Coupled Model

**Figure 10:**
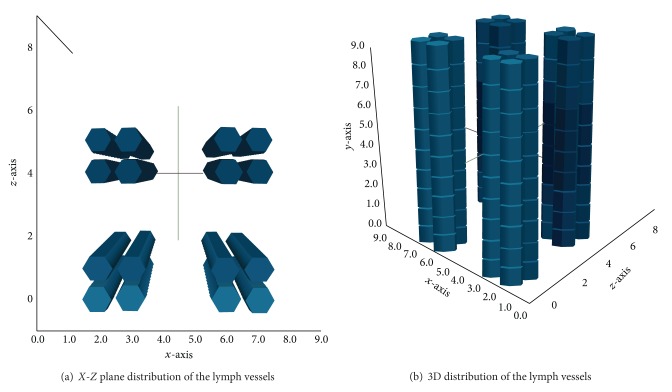
Scheme representing the position of the lymph vessels in the tissue. They are not centralized to better visualization of the cells diffusion.

The third case represents the complete scenario with the APCs stimulating the lymphocytes. The coupled model scenario was simulated considering four blood capillaries and four lymph capillaries inside the 10 mm^3^ cubic domain. The capillaries were placed, respectively, on the edges of the cube over the *y*-axis (Figure 7(b)) and distributed near the central portion of the cubic domain over the same *y*-axis (Figures 10(a) and 10(b)).

**Figure 11:**
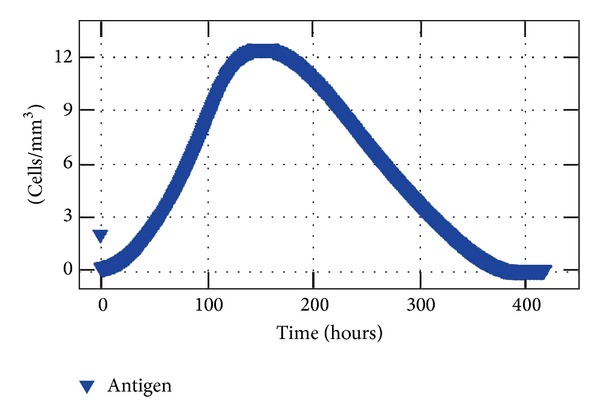
Antigen concentration (average) in the tissue for 20 days of simulation of the coupled model.

This distribution is given by a subroutine which is easily modified to allow distinct configurations. The antigen initial condition is the same simulated on the second case: an injection in the central portion of the domain. A 30-day simulation was performed and the results showing the coupling follow within this section. With the arrival of antibodies in the tissue the immune response was able to eliminate the antigen after 20 days of simulation as shown in Figure 11.

**Figure 12:**
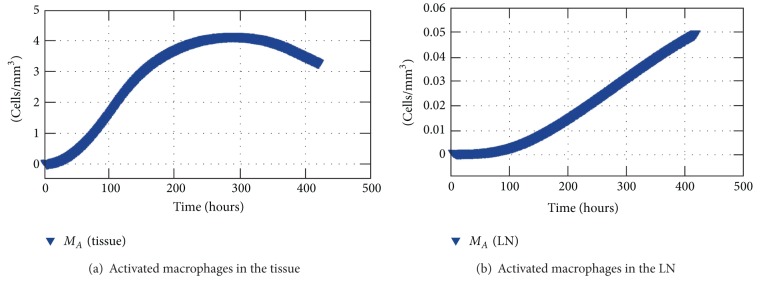
Average concentration of activated macrophages in the tissue and concentration of activated macrophages in the nearest LN.

Figure 12 shows a comparison between the average population of activated macrophages inside the tissue and the population of activated macrophages in the LN. Logarithmic scale was used for the comparison. As one can observe, activated macrophages are migrating to the nearest LN to work as APCs: as the resting macrophages become activated, their population increases in the tissue as well as in the LN, which shows that the coupling is working.

**Figure 13:**
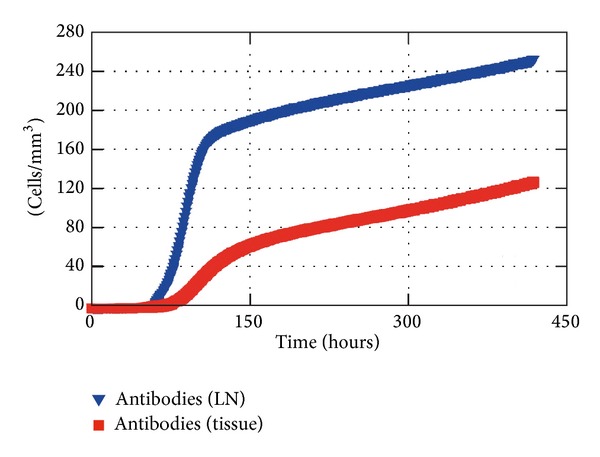
Antibodies in the LN and in the tissue (average) during 20 days of simulation.

Other feature of the coupling is the migration of antibodies produced after the stimulation cascade from the LN to the location where the infection takes place. The concentration of antibodies is shown in Figure 13 in which an average of antibodies inside the tissue is represented over time as well as the concentration of antibodies in the nearest LN. It can be observed that the antibodies start to be produced early in the simulation, just after a few hours, what is due to the presence of activated macrophages in the LN. A small amount of antibodies migrate to the local of the inflammation through the blood vessels after a few hours and a significant amount is present in the tissue after a few days which contributes to the elimination of antigens.

The average of antibodies, as well as the average of macrophages, tends to a steady state instead of decreasing after the elimination of the antigen. This happens due to the absence of a self-regulation process. At the moment, as the macrophages that are activated are still present in the LN, they continue to stimulate the lymphocytes to produce more antibodies which keep migrating to the tissue. However, the acquired response is self-regulated and we expect to add this feature to the model through the addition of anti-inflammatory cytokines, such as IL-4 or IL-10. This work is already in progress.

**Figure 14:**
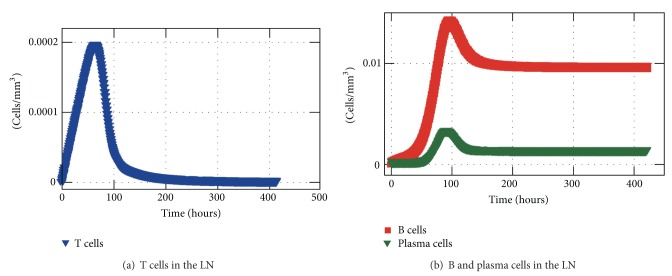
T-lymphocyte, B-lymphocyte, and plasma cell concentrations in the LN.

Figure 14 shows the concentration of T-Lymphocytes, B-Lymphocytes, and plasma cells, within 20 days of simulation. It could be noticed that the activation is happening early with the increase of lymphocytes in the first hours. Thus, the peak of those cells occurs approximately after 2 days for the T-lymphocytes and around the 5th day for B-lymphocytes and plasma cells.

**Figure 15:**
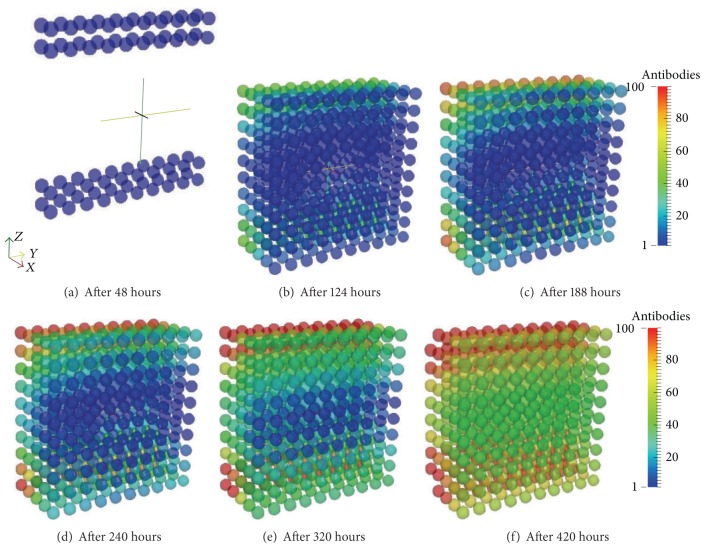
Antibodies arriving in the tissue through the blood vessels. Initially the antibodies do not exist in the tissue and they arrive through the capillaries and start to diffuse over the tissue.

**Figure 16:**
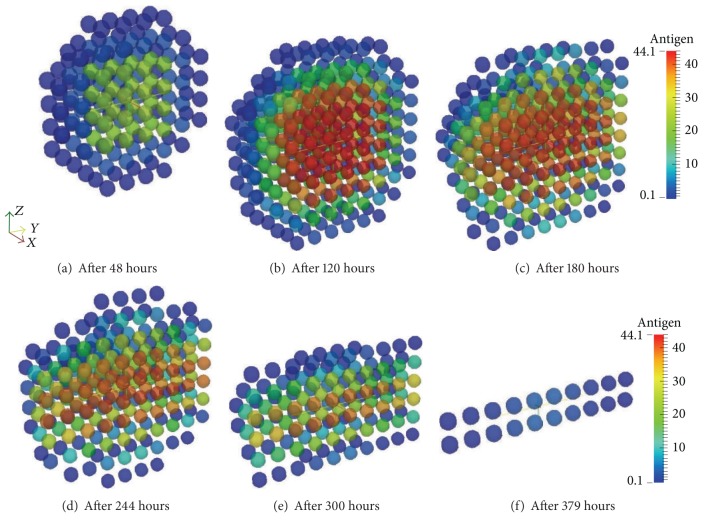
Antigen diffusion during 20 days of simulation of the coupled model. The macrophages are capable of restraining antigen growth but they are not able to defeat this initial amount. However, the antibodies arriving through the blood vessels help macrophages to eliminate the antigen through opsonization.

Aiming to show the diffusive process and the effect of the arrival of the antibodies in the tissue the set of Figures 15(a)–15(f) presents the antibodies and Figures 16(a)–16(f) present the antigens. The antibodies arrive at the tissue through the lymph vessels which are positioned according to Figures 10(a) and 10(b). The domain is a hexahedron which was sliced to better visualization henceforth there are 4 vessels but Figures 15(a)–15(f) only show half of them. Moreover, Figures 16(a)–16(f) are also sliced to better visualization of the diffusion.

After a couple of days of the beginning of the simulation, it is possible to see the antigen starting to diffuse (Figure 16(a)). The antigen continues to diffuse slowly restrained by the innate response until approximately the 3rd day. After a few days the amount of antibodies that is arriving at the tissue helps macrophages to defeat the antigen more efficiently in the regions where there is more concentration of antibodies (Figures 16(c)–16(f)).

### 4.4. Sensitivity Analysis of the Coupled Model

The sensitivity analysis can be used to help with the verification of a mathematical model by evaluating how the model responds to changes in one or more inputs. The validation of the model involves comparison of the results to independent observations from the system being modeled which is not always feasible. Therefore, the sensitivity analysis can be used to understand the behavior of the model and reach a comfortable position in terms of qualitative results [66]. Thus, there are several ways of performing this assessment of the sensitivity of the model; it was chosen herein the so called one-factor-at-a-time approach (OAT), which is the most used strategy [67].

The sensitivity analysis of the coupled model considered the complete scenario during 30 days in a cubic centimeter domain. Each chosen parameter on Table 5 was assessed one at a time varying its value from −100% to +200% to understand its influence on the output. Table 5 presents the chosen parameters with a brief description and their maximum error value (Max_*err*⁡_).

The error was calculated applying (15) to each parameter as follows:

(15) $$\begin{array}{c} \text{Ma}{\text{x}}_{\mathrm{err}}={\text{MAX}}_{k}\left(\frac{\sqrt{\sum_{i=0}^{N}{\left(E_{\text{orig}}\left(i\right)-E_{k}\left(i\right)\right)}^{2}}}{\sqrt{\sum_{i=0}^{N}{\left(E_{\text{orig}}\right)}^{2}}}\right), \end{array}$$

in which *k* index each variation within the same parameter. *E*_orig_ is the number of antigens over time using the original set of parameters, *E*_*k*_ represents each resultant number of antigens over time with the variation of one parameter at-a-time and *N* is the number of time steps. Thus, we have a maximum error value for each parameter that enables us to understand which parameters are the most sensitive of the coupled model.

**Figure 17:**
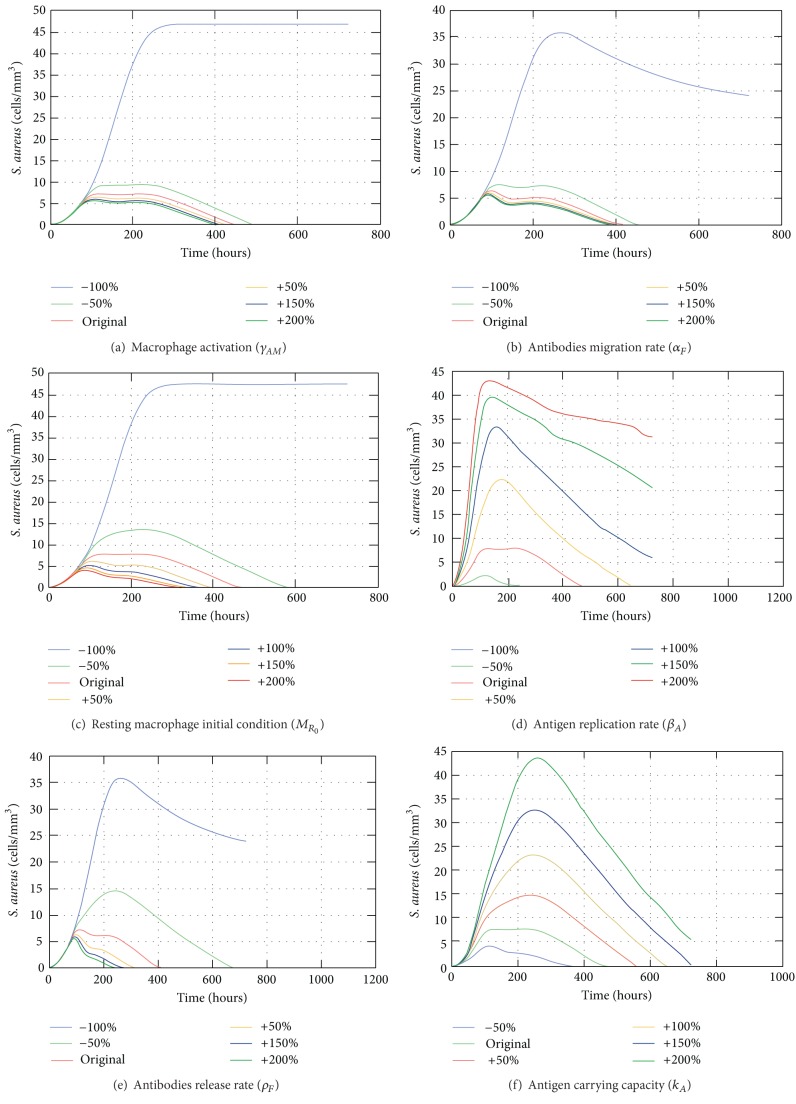
Impact of the variation of *γ*_AM_,*α*_*F*_, *M*_*R*_0__, *β*_*A*,_ *ρ*_*F*_ and *k*_*A*_ on the population of antigens.

Among the 20 assessed parameters we are going to consider those with Max_*err*⁡_ ≥ 1. Therefore, the most sensitive parameter is the rate of activation of macrophages *γ*_AM_. Without the activation, the acquired response is not triggered and the response depends only on the resting macrophages (Figure 17(a)). The second one is the migration rate of the antibodies to the tissue (*α*_*F*_). This parameter is essential to the acquired response and without this migration rate there are only resting and activated macrophages in the tissue (Figure 17(b)). The initial condition of resting macrophages (*M*_*R*_0__) is also significant for the model by the fact that without them there is practically no response as they are responsible for recognizing and engulfing the antigen (Figure 17(c)). After they identify the antigens they become APCs which triggers the acquired response. According to the results shown in Figure 17(d), without the replication rate (−100%) the amount of antigen in the tissue remains reduced but sufficient to trigger the immune response which eliminates the antigen. Increasing this rate, the immune response as a whole takes more time to eliminate the antigen. With more than 50% of increase the response is not able to defeat the antigen in 30 days. The release rate of antibodies in the LN (*ρ*_*F*_) also affects directly the acquired response. As it can be observed in Figure 17(e), doubling the original value the antigens are eliminated in almost half the time (approximately 9 days) whereas decreasing this rate by 50% the response takes approximately 28 days. The carrying capacity parameter, shown in Figure 17(f), is the only one that cannot be varied from −100%; otherwise it would generate a division by zero (1). So, it was varied from −50% and for that value, the response is able to eliminate the antigen a couple of days before the original value used in simulations, whereas doubling the value, it takes approximately more 10 days to defeat the antigen. If there is more antigen that is able to replicate the immune response needs more time to defeat them.

**Figure 18:**
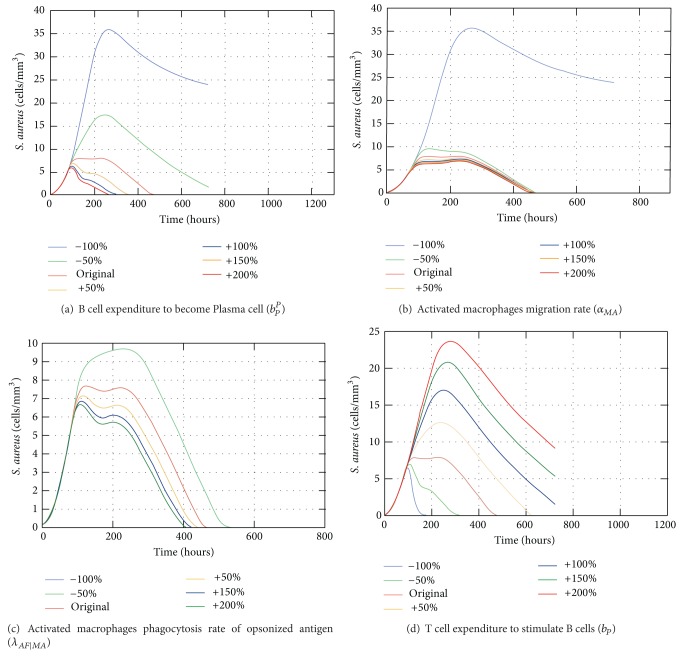
Impact of the variation of *b*_*P*,_^*P*^ *α*_*MA*_,*λ*_*AF*|*MA*_ and *b*_*P*_ on the population of antigens.

The coefficient *b*_*P*_^*P*^ is important to determine the number of B cells that turn into plasma cells. If this value is reduced to 100% it means that not a single B cell turns into a plasma cell leading to a nonexistent specific response. However, increasing this parameter leads to larger production of antibodies and quicker specific response to eliminate antigens (Figure 18(a)). Moreover, the activated macrophage migration rate to the LN (*α*_*MA*_) is essential to initiate the acquired response as without this migration the antigen is not presented to the lymphocytes in the LN and the specific antibodies are not mass produced. Thus, if this parameter is set to zero there is only the innate response (Figure 18(b)). The activated macrophages phagocytosis rate of opsonized antigen, *λ*_*AF*∣*MA*_, also shows great discrepancy if set to zero (Figure 18(c)). This rate is important to the effectiveness of the acquired response and the more its value is increased the earlier the response is able to eliminate the antigens. Setting the coefficient *b*_*p*_ equal to zero means that the B cells are stimulated even without the presence of T cells, leading to the constant stimulation of existent B cells in the LN. Those B cells turn into plasma cells which population increases promptly in the LN. Also, as soon as the activated macrophages arrive in the LN, a large production of antibodies starts. As a consequence, a great number of antibodies arrive in the tissue, approximately 4 days after the injection of antigens. Approximately 3 days after the arrival of the antibodies the antigens are eliminated. Meanwhile, if the value of that coefficient is 200% bigger, it means that a lot more T cells are needed to stimulate the B cells, leading to a much smaller number of antibodies arriving in the tissue, which in turn are not able to help macrophages to defeat the antigens in 30 days (Figure 18(d)).

### 4.5. Discussion

Based upon two distinct mathematical models of the human immune system a novel form of coupling models was developed. This coupling was performed firstly by analyzing the features of each previous model to identify the possible bridges between them and then building the linking itself. The choice of coupling models instead of developing a whole new set of equations is due to the fact that these models were already validated experimentally, which is not easy to achieve without collaborative work. We chose a model with already fitted parameters [44] and we had to convert those parameters to the unit we were using in previous work [42] as the former units were given in molar concentration (mol) and we expect to analyse the number of interactive cells per cubic millimeter.

In order to perform the coupling we had to add some terms to the PDEs and even a new Equation (4) to represent the interactions between the cells and their migration. The former PDEs based model [42] was not able to simulate the presence of antibodies inside the tissue which was achieved with the coupling performed in this work. That model represents other features of the immune response as the presence of neutrophils and the chemotaxis process which are not present in this coupling yet due to simplification. Our intention is to focus on the coupling and then improve the model with those characteristics of the innate IS. The terms that were added to the model are the ones which control the flux of cells between the tissue and the nearest LN (3) and the terms that represent the opsonization process in (1).

The former ODEs based model [44] actually represented some features of the acquired response such as the clonal expansion of T helper cells with delay differential equations (DDEs). DDEs have been used to model biological processes as they give a better approximation for such aspects [68, 69]. We have opted not to use DDEs initially in order to simplify the model as we solve the equations using our own solver. We hope to introduce this concept in the future to better represent those biological processes. We modified that model in the following aspects: (a) we removed the equation for bacteria equilibrium due to the fact that we are representing this insertion of bacteria inside the tissue (modeled by the PDE given by (1)) and (b) we modified the equation that represents the antigen presenting cells in the LN (5). The previous equation considered bacteria stimulation and natural decay. The modified equation solely considers the flux of active cells between the tissue and the LN as the activation and natural decays are represented locally in the tissue (3). This representation required an integration of the concentration of cells in the tissue to estimate the amount of cells in the LN (7).

We would like to reinforce that those two models presented herein were chosen due to the availability of parameters already fitted. The outcomes of the coupled model agree qualitatively with the literature [44]. Further quantitative validation of the coupling is still required. That could be achieved, for example, by comparing the outputs of the coupled model to results obtained experimentally. This is an ongoing work. We would also like to perform this coupling with other models to gain insights into a specific infection scenario.

## 5. Conclusions

This work presented the coupling of two distinct models of different aspects of the immune system: one of them uses PDEs to model the dynamics of cells in a three-dimensional section of tissue and the other one uses ODEs to model the dynamics of cells in the nearest LN. To the best of our knowledge, the integration of two models in the format presented has not been proposed before in the field of immune systems. To exemplify the coupling, a mathematical and computational coupling of models was presented that simulates the immune response to* S. aureus* bacteria into a three-dimensional section of a tissue. To achieve this goal, the models reproduce the initiation, maintenance, and resolution of innate and adaptive immune response. A set of PDEs and ODEs are used to model the main agents involved in this processes, like the antigen, macrophages, antibodies, and T and B cells.

The model presented in this work represents an infection scenario: the diffusion of antigens into the tissue and the migration of macrophages to combat the infection. Macrophages also migrate outside the tissue and stimulate the adaptive IS to produce antibodies, which in turn migrate inside the tissue and opsonize the antigens. The proposed integrated model was capable of reproducing qualitatively the spatial and temporal behavior of resting and activated macrophages as well as specific antibodies.

A sensitivity analysis was performed for the coupled model showing that the most relevant parameters are the ones related to the activation of the response, as the macrophage activation rate (*γ*_*AM*_), effectiveness of the acquired response as the antibodies migration rate (*α*_*F*_), and the presence of the immune responses itself as the resting macrophage initial condition (*MR*_0_). Other parameters that are important to the success of the immune response are the antigen replication rate (*β*_*A*_), antibodies release rate (*ρ*_*F*_), and the amount of antigens that could grow in the tissue (*k*_*A*_).

We expect that with that spatial coupled model we could simulate and analyze the evolution of damages caused to an organ parenchyma, for example, the damage in the lung tissue caused by tuberculosis or pneumonia. Also, we are already implementing a more complete mathematical model including molecules, like cytokines, and others processes involved in the immune responses to consider the chemotaxis process. Furthermore, we intend to improve the visualization of the damage caused to the tissue in order to compare to medical imaging results. Thus, we believe that the coupling of models in the proposed format could provide some insight into the behavior of the immune system.

## Figures and Tables

**Algorithm 1:**
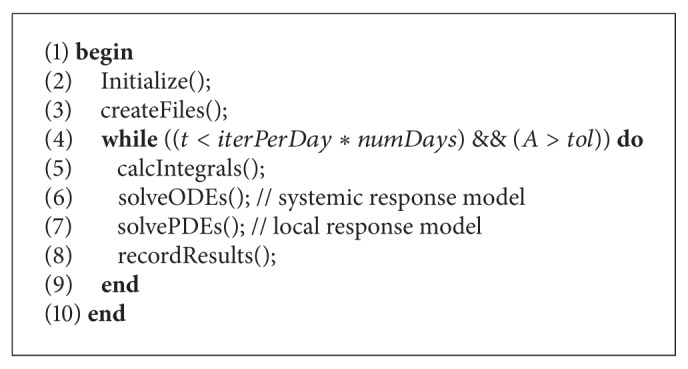
Main program for the coupled models.

**Table 1:** Initial values of the coupled model.

Parameter	Value	Unit	Reference
*A*_0_	2	Cell/mm^3^	Estimated
*M*_*R*_0__	4	Cell/mm^3^	Estimated
*M*_*A*_0__	0.0	—	[44]
*F*_0_	0.0	—	[44]

*T*_0_	0.0	—	[44]
*B*_0_	0.0	—	[44]
*P*_0_	0.0	—	[44]
*F*_0_	0.0	—	[44]
T*	8.4∗10^−3^	Cell/mm^3^	[44]
B*	8.4∗10^−4^	Cell/mm^3^	[44]
P*	8.4∗10^−6^	Cell/mm^3^	[44]
F*	0.0	Cell/mm^3^	[44]
MR*	4	Cell/mm^3^	Estimated

**Table 2:** Diffusion coefficients.

Parameter	Value	Unit	Reference
*D*_*A*_	3.7∗10^−5^	mm^3^/day	[62]
*D*_*MR*_	4.32∗10^−2^	mm^3^/day	[42]
*D*_*MA*_	0.3	mm^3^/day	[42]
*D*_*F*_	1.6∗10^−2^	mm^3^/day	[42]

**Table 3:** Replication, decay, activation, and phagocytosis rates.

Parameter	Value	Unit	Reference
*β*_*A*_	2.0	1/day	[44]
*k*_*A*_	50.0	cell/mm^3^	Estimated

*μ*_*A*_	0.1	1/day	[44]
*μ*_*MR*_	0.033	1/day	[42]
*μ*_*MA*_	0.07	1/day	[42]

*γ*_*AM*_	8.3∗10^−2^	mm^3^/cell∗day	[44]

*λ*_*MR*_	5.98∗10^−3^	mm^3^/cell∗day	[44]
*λ*_*MA*_	5.98∗10^−2^	mm^3^/cell∗day	[44]
*λ*_*AF*∣*MR*_	1.66∗10^−3^	mm^6^/cell^2^∗day	[44]
*λ*_*AF*∣*MA*_	7.14∗10^−2^	mm^6^/cell^2^∗day	[44]

**Table 4:** Other coefficients used in the coupled model.

Parameter	Value	Unit	Reference
*α*_*MA*_	10^−3^	1/day	[44]
*α*_*T*_	0.01	1/day	[44]
*α*_*B*_	1.0	1/day	[44]
*α*_*P*_	5.0	1/day	[44]
*α*_*F*_	0.43	1/day	[44]
*α*_*MR*_	4.0	1/day	Estimated

*b*_*T*_	1.7∗10^−2^	mm^3^/cell∗day	[44]
*b*_*P*_	10^5^	mm^6^/cell^2^∗day	[44]
*b*_*P*_^*B*^	6.02∗10^3^	mm^6^/cell^2^∗day	[44]
*b*_*P*_^*P*^	2.3∗10^6^	mm^3^/cell∗day	[44]

*ρ*_*T*_	2.0	—	[44]
*ρ*_*B*_	16.0	Cell/mm^3^	[44]
*ρ*_*P*_	3.0	—	[44]
*ρ*_*F*_	5.1∗10^4^	—	[44]
*V*_LN_	160	Cells	Estimated

**Table 5:** Sensitivity analysis—chosen parameters, description, and the maximum error value.

Parameter	Description	*Max*⁡_*err*⁡_⁡
*γ*_*AM*_	Macrophage activation rate	6.47

*α*_*F*_	Antibodies migration rate	6.36

*M*_*R*_0__	Resting macrophage initial condition	6.17

*β*_*A*_	Antigen replication rate	5.67

*ρ*_*F*_	Antibodies release rate	5.15

*k*_*A*_	Antigen carrying capacity coefficient	4.82

*b*_*P*_^*P*^	B cell expenditure to become plasma cell	4.09

*α*_*MA*_	Activated macrophage migration rate	4.09

*λ*_*AF*∣*MA*_	Activated macrophage phagocytosis rate of opsonized antigen	4.06

*b*_*P*_	T cell expenditure to stimulate B cell	2.42

*D*_*MR*_	Resting macrophage diffusion coefficient	0.93

*D*_*A*_	Antigen diffusion coefficient	0.85

*λ*_*MA*_	Activated macrophage phagocytosis rate	0.76

*b*_*P*_^*B*^	B cell stimuli coefficient	0.73

*D*_*MA*_	Activated macrophage diffusion coefficient	0.24

*A*_0_	Antigen initial condition	0.09

*α*_*MR*_	Resting macrophage source coefficient	0.07

*λ*_*MR*_	Resting macrophage phagocytosis rate	0.05

*λ*_*AF*∣*MR*_	Resting macrophage phagocytosis rate of opsonized antigen	0.01

*b*_*T*_	T cell stimuli coefficient	3∗10^−4^
